# Direct Discrimination and Growth Estimation of Foodborne Bacteria in Raw Meat Using Electronic Nose

**DOI:** 10.3390/microorganisms12112250

**Published:** 2024-11-07

**Authors:** Wellington Belarmino Gonçalves, Wanderson Sirley Reis Teixeira, Aryele Nunes da Cruz Encide Sampaio, Otávio Augusto Martins, Evelyn Perez Cervantes, Mateus de Souza Ribeiro Mioni, Jonas Gruber, Juliano Gonçalves Pereira

**Affiliations:** 1 Departamento de Química Fundamental, Instituto de Química, Universidade de São Paulo, Av. Prof Lineu Prestes, 748, São Paulo 05508-000, SP, Brazil; wellington2906@gmail.com (W.B.G.); jogruber@iq.usp.br (J.G.); 2 Faculdade de Medicina Veterinária e Zootecnia, Universidade Estadual Paulista “Júlio de Mesquita Filho” (UNESP), Botucatu 18618-681, SP, Brazil; wanderson.teixeira@unesp.br (W.S.R.T.); aryele.sampaio@unesp.br (A.N.d.C.E.S.); otavio.a.martins@unesp.br (O.A.M.); 3 Instituto de Matemática e Estatística, Universidade de São Paulo, São Paulo 05508-090, SP, Brazil; epcervantes7@gmail.com; 4 Departamento de Patologia, Reprodução e Saúde Única, Faculdade de Ciências Agrárias e Veterinárias, Universidade Estadual Paulista “Júlio de Mesquita Filho” (UNESP), Jaboticabal 14884-900, SP, Brazil; mateus.mioni@unesp.br

**Keywords:** microbiology, foodborne bacteria, meat, food safety, electronic nose, machine learning

## Abstract

Evaluation concerning the presence of bacteria in meat products is mandatory for commercializing these goods. Although food bacteria detection is based on microbiological methods, these assays are usually laborious and time-consuming. In this paper, an electronic nose is used to differentiate *Salmonella* spp. (SA), *Escherichia coli* (EC), and *Pseudomonas fluorescens* (PF) inoculated in raw meat (beef, chicken, and pork) and incubated at 22 °C for 3 days. The obtained data were evaluated by principal component analysis (PCA) and different machine learning algorithms. From the graphical analysis of the PCA, on day 1, the clusters were close to each other for beef, chicken, and pork, while on days 2 and 3, more separated bacteria clusters were obtained regardless of the meat type, allowing for the discrimination of the samples for the latter days. To estimate the growth rates of the microorganisms, the distance between clusters was calculated and provided a pattern for the three bacteria, with the slowest-, moderate-, and fastest-growing being EC, SA, and PF, respectively. Concerning the machine learning algorithms, the accuracy varied from 93.8 to 100% for beef and chicken, while for pork, it varied from 75% to 100%. Thus, these results suggest that the proposed methodology based on electronic nose has the potential for the direct discrimination of bacteria in raw meat, with reduced analysis time, costs, and manipulating steps.

## 1. Introduction

Food security, a term that defines the physical, social, and economic access to sufficient, safe, and nutritious food for a healthy and effective life [1], is a global concern and a priority in terms of achieving world welfare, being specially addressed in Sustainable Development Goal number 2 (zero hunger), made by the United Nations. Around 30% to 40% of the food produced is spoiled or wasted in the supply chain before reaching the final customer due to microbiological, physical, or chemical reasons, with spoilage and pathogenic microbes being the most common cause [1,2,3]. For example, diarrheal diseases are the second leading cause of death in children under five years old worldwide, accounting for the death of about hundreds of thousands of children every year. In addition, diarrhea is one of the leading causes of malnutrition, making people more susceptible to other diseases. Diarrhea is usually a symptom of an intestinal infection that can be caused by various bacterial, viral, and parasitic organisms [4]. Therefore, foodborne diseases limit food production and distribution, contributing directly and indirectly to global food insecurity [5,6].

In the meat industry, the detection of spoilage and pathogenic bacteria is necessary. Depending on the type of meat product, international standards and Brazilian legislation require the analysis of *Salmonella* spp., *Escherichia coli*, *Clostridium perfringens*, *Staphylococcus* spp., and other microorganisms [7,8,9]. In addition, *Pseudomonas* spp. is also usually evaluated since it is an important spoilage agent, and due to its high incidence in the industry [10,11].

Currently, the evaluation of bacterial contamination in food quality control is mostly based on microbiological assays [12], and despite all the developments in microbiology in recent decades, the usual techniques have several drawbacks, such as laborious procedures, long analysis times, and contamination risks [13]. These disadvantages can be overcome by some modern analytical strategies, such as matrix-assisted laser desorption/ionization-time of flight (MALDI-TOF) [14,15], polymerase chain reaction (PCR) [16], genetic sequencing combined with bioinformatics [17,18], and different microscopic techniques [19,20]. However, these techniques are expensive and require specialized professionals and infrastructure, making their use in routine laboratories unaffordable [13].

Since microbial organisms generate specific volatile organic compounds (VOCs) as by-products of their natural metabolism, this complex mixture of gasses can be used to differentiate microorganisms using an electronic nose (e-nose), a user-friendly and cheap methodology, by establishing a standard profile related to these VOCs. This concept has been applied to analyze fungi and bacteria in fruits, vegetables, beverages, meats, and other processed foods before or after microbial enrichment [21,22,23].

In a comprehensive review, Casaburi et al. described the relationship between bacterial populations and the VOCs associated with meat spoilage, raw meat aroma molecules, and spoilage-associated sensory implications [24]. In this work, the authors presented data about some of the most commonly identified VOCs related to bacteria growth in fresh meat, including the following compounds: alcohols, esters, ketones, aldehydes, sulfur compounds, amines, and volatile fatty acids [24].

Focusing on the use of an e-nose as a tool for the evaluation of quality in meat products due to bacteria spoilage, Balasubramanian et al. developed a method to predict the population of *Salmonella* Typhimurium in beef. For this work, meat samples were inoculated with the bacteria, and VOCs were exposed to an e-nose for five days. Analyzing the data generated by the device combined with statistical techniques, it was possible to develop a method to predict the concentration of bacteria in the meat at different temperatures, achieving an accuracy of 69% at 4 °C and above 80% at 10 °C [25]. Ramírez et al. assessed the shelf life of fresh pork with a commercial e-nose using physicochemical, sensory, and microbiological parameters. The response of the e-nose was qualitatively validated and significantly correlated with the sensory attributes, total biogenic amine content, and microbial counts [26].

Astuti et al. demonstrated the application of an electronic nose based on commercial gas sensors for comparing fresh chicken meat and chicken meat contaminated with *E. coli*. For this experiment, the authors evaluated the variance in the sensors’ voltage as input data for classifier algorithms: random forest and support vector machine. For the optimized conditions, the fresh and contaminated samples were classified with a precision of 99.25% and 98.42%, respectively [27]. Damdam and coworkers presented a versatile Internet of Things (IoT)-enabled electronic nose system to monitor food quality by evaluating the concentrations of VOCs (carbon dioxide, ammonia, and ethylene) and used the system for identifying beef spoilage stored at 4 °C and 21 °C. The authors correlated the production of VOCs with the proliferation of bacteria using linear regression, and suggested that aerobic bacteria and *Pseudomonas* spp. play a significant role in the production of VOCs in raw beef, as opposed to lactic acid bacteria [28].

The feasibility of the rapid and nondestructive evaluation of pork freshness using a portable e-nose based on a colorimetric sensor array was proposed by Li et al. The low-cost sensor array was fabricated by printing 12 chemically responsive dyes on a silica-gel flat plate with a specific color fingerprint for the volatile compounds released from the pork samples. Under optimized conditions, the total analysis time was 5 min, and the discrimination rates were 100% and 97.5% for the training and prediction sets, respectively, demonstrating the potential of this technology for the real-time monitoring of meat quality [29]. In other work, Gu et al. developed a method for predicting the growth of *Pseudomonas aeruginosa* based on the generated VOCs on agar plates and meat pieces using an e-nose composed of 10 sensors. Then, optimal sensors were selected to simulate the bacteria’s growth using modified Logistic and Gompertz equations. The results showed a correlation between the models and *P. aeruginosa* growth on an agar plate and for inoculated meat stored at 4 °C and 20 °C, suggesting the use of their gas sensors as a rapid, easy, and nondestructive method for predicting the bacteria’s growth [30].

In this sense, this study aimed to evaluate the application of an e-nose combined with PCA and different machine learning algorithms to differentiate *Salmonella* spp., *Escherichia coli,* and *Pseudomonas fluorescens* inoculated directly into meat samples of beef, chicken, and pork as a possible fast indicator of food innocuity and deterioration.

## 2. Materials and Methods

### 2.1. Chemicals and Bacterial Strains

1-Ethyl-3-methylimidazolium dicyanamide ionic liquid (EMIMDCA, purity > 98%) and bovine skin gelatin type B were purchased from Sigma Aldrich (Saint Louis, MO, USA). Tetrahydrate iron (II) chloride (FeCl_2_·4H_2_O, purity > 99%) was obtained from Vetec (Duque de Caxias, RJ, Brazil). Iron (III) chloride hexahydrate (FeCl_3_·6H_2_O, purity ≥ 99%) was obtained from Acros Organic (Geel, Antwerp, Belgium). Ammonium hydroxide was purchased from Labsynth (Diadema, SP, Brazil). Brain Heart Infusion (BHI) medium was obtained from Basingstoke, Hampshire, UK. The interdigitated electrodes were manufactured by Micropress S.A. (São Paulo, SP, Brazil) with a 0.6 cm^2^ interdigitated area, 200 µm of spacing between the copper digits, and 100 µm of spacing between the nickel digits covered with 0.05 µm of gold.

The meat samples were from knuckle (beef), breast filet (chicken), and ham (pork) obtained from commercial brands available in supermarkets (Botucatu, SP, Brazil). All bacterial strains used in this study were ATCC-standard, namely, *Salmonella* Typhimurium (ATCC 14028—SA), *Escherichia coli* (ATCC 8739—EC), and *Pseudomonas fluorescens* (ATCC 13525—PF).

### 2.2. Raw Meat Inoculation

The bacteria activation was conducted in BHI for 24 h at 37 °C. For the raw meat inoculation, 1 mL of BHI suspension (2 log CFU·mL^−1^) previously prepared for each bacterium was transferred to a Falcon tube containing 5 g of the respective meat. The tubes were incubated at 22 °C, and e-nose measurements were collected before the bacteria inoculation and after 1, 2, and 3 days. The temperature of 22 °C was used to induce a restriction process and favor bacterial multiplication.

### 2.3. Sensors’ Preparation and E-Nose Equipment

The sensors used in this study were obtained according to those from our previous works [31,32]. Briefly, four metallic interdigitated electrodes were covered by an ionogel composed of bovine gelatine (75 µL), EMIMDCA (30 mg), and Fe_3_O_4_ particles at different concentrations (0, 25, 50, and 75 mg·mL^−1^). The conductance of each sensor was registered by a conductivity meter.

The e-nose equipment (São Paulo, SP, Brazil) and data acquisition software (Nose_v7) were lab-made and previously described [33,34]. Succinctly, the software controlled the gas valves and measured conductance. During the cleaning (recovery) step, the air pump drove ambient air directly to the sensors’ chamber. In contrast, during the exposition step, VOCs from the sample chamber were driven to the sensors by the air. Based on our previous studies, the ionogel’s sensors presented stable signals over several cycles [35] and did not require any additional steps for cleaning or preparation between sequential samples [36].

### 2.4. Data Acquisition

In this study, 8 cycles of exposition (5 s) and recovery (150 s) were used, corresponding to the total analysis time of approximately 21 min. Aiming to obtain a stable baseline and avoid signal variation due to the influence of VOCs left from the previous sample, the first 4 cycles were not used throughout this work, and only the latter measurements were considered for each given sample.

For each meat sample inoculated with the three different bacteria, measurements were taken for days 1, 2, and 3. Besides that, the mean data of relative responses (RRs) taken for beef, chicken, and pork meat before bacteria contamination (day 0) were also taken and used for data normalization and as an internal reference, allowing for the comparative evaluation among the different experimental conditions.

### 2.5. Data Treatment

The data treatment approaches used in this work were previously described [31,32]. Briefly, PCA was used as a graphical analysis tool (OriginPro 2018, OriginLab Coorporation, Northampton, MA, USA), while linear discriminant analysis (LDA), instance-based (IBk), and Logistic Model Tree (LMT) were evaluated as machine learning algorithms for automated classification (Weka 3.8, University of Waikato, Hamilton, New Zealand) [37]. For all automated classifiers, k-fold was used as a cross-validation method, k = 10.

To estimate information about the bacteria growing pattern based on the PCA biplot obtained from the mean values of normalized RR data, the distance between a given point to the blank was calculated according to Equation (1).

(1) $$distance=\sqrt{{\left(x_{dayn}-x_{blank}\right)}^{2}+{\left(y_{dayn}-y_{blank}\right)}^{2}}$$

where *x_dayn_* and *y_dayn_* are the coordinates for a given sample, while *x_blank_* and *y_blank_* are the coordinates related to the meat on day 0.

## 3. Results

### 3.1. E-Nose General Data

**Figure 1:**
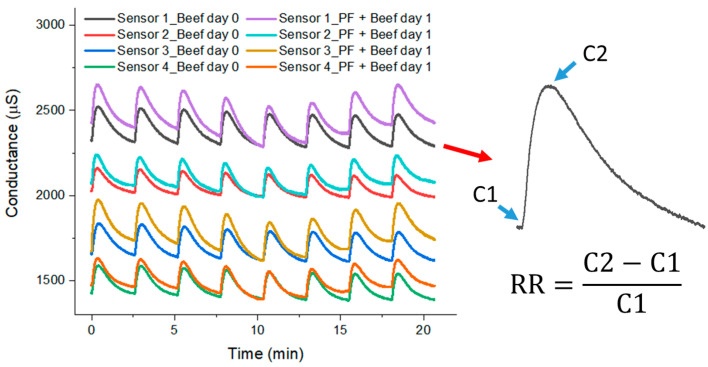
Response of the sensors when exposed to beef without bacteria (day 0) and beef inoculated with PF (day 1). Inset corresponds to RR calculation.

For illustrating the general response of the electronic nose, the curves of conductance × time for the four different sensors are presented in Figure 1, considering beef before contamination (day 0) and beef inoculated with PF (day 1) as samples. The conductance data make it possible to calculate the RR for each sensor individually during each cycle (inset in Figure 1). As mentioned above, only the latter four cycles of exposition and recovery were considered for the sample discrimination, and their normalized RR values are presented in Supplementary File Table S1.

### 3.2. Bacteria Discrimination

**Figure 2:**
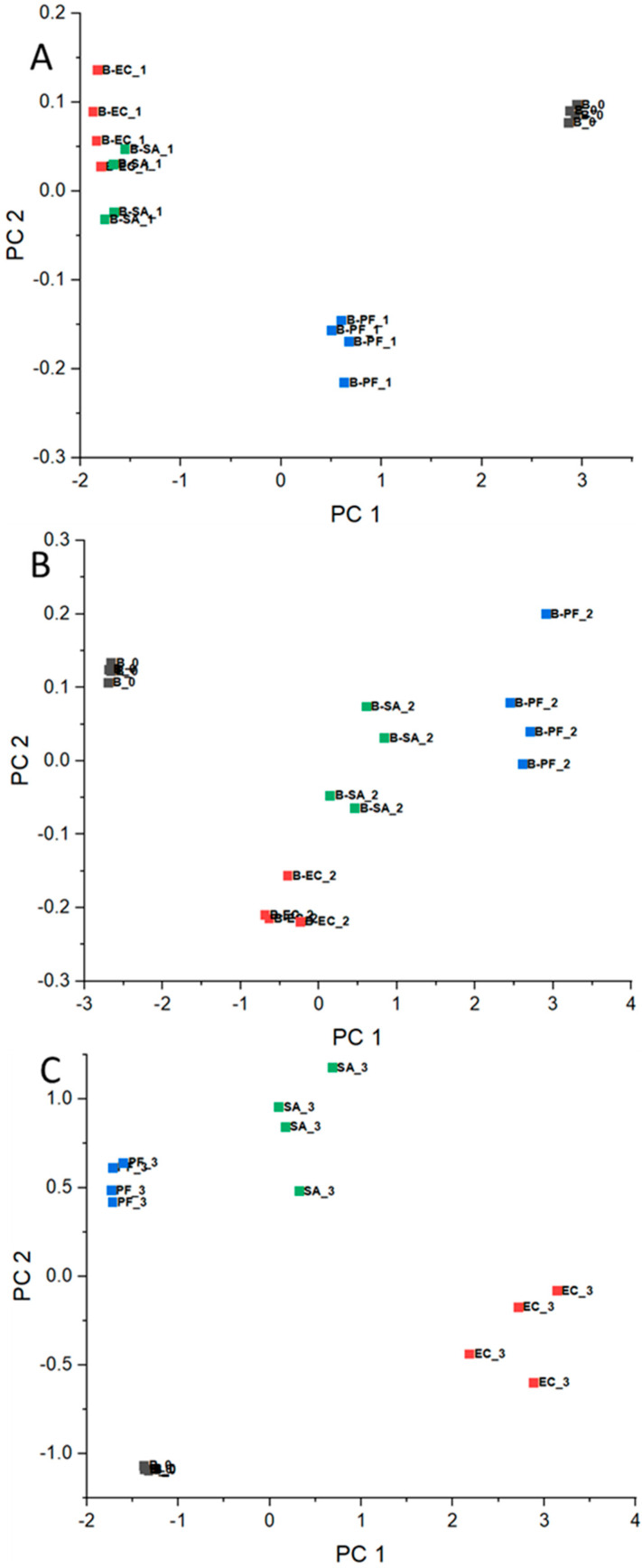
PCA for beef meat before contamination (day 0—■) and inoculated with EC—■, PF—■, and SA—■ for days (**A**) 1, (**B**) 2, and (**C**) 3.

**Figure 3:**
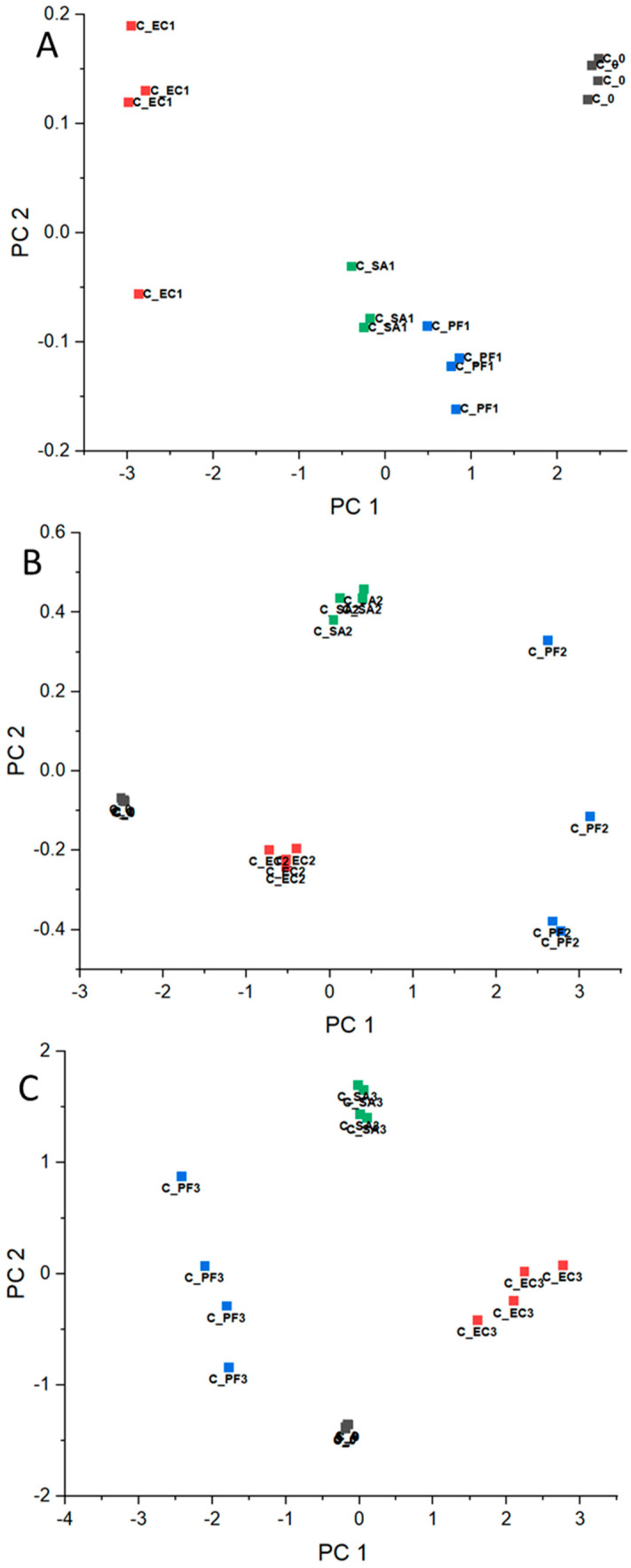
PCA for chicken meat before contamination (day 0—■) and inoculated with EC—■, PF—■, and SA—■ for days (**A**) 1, (**B**) 2, and (**C**) 3.

**Figure 4:**
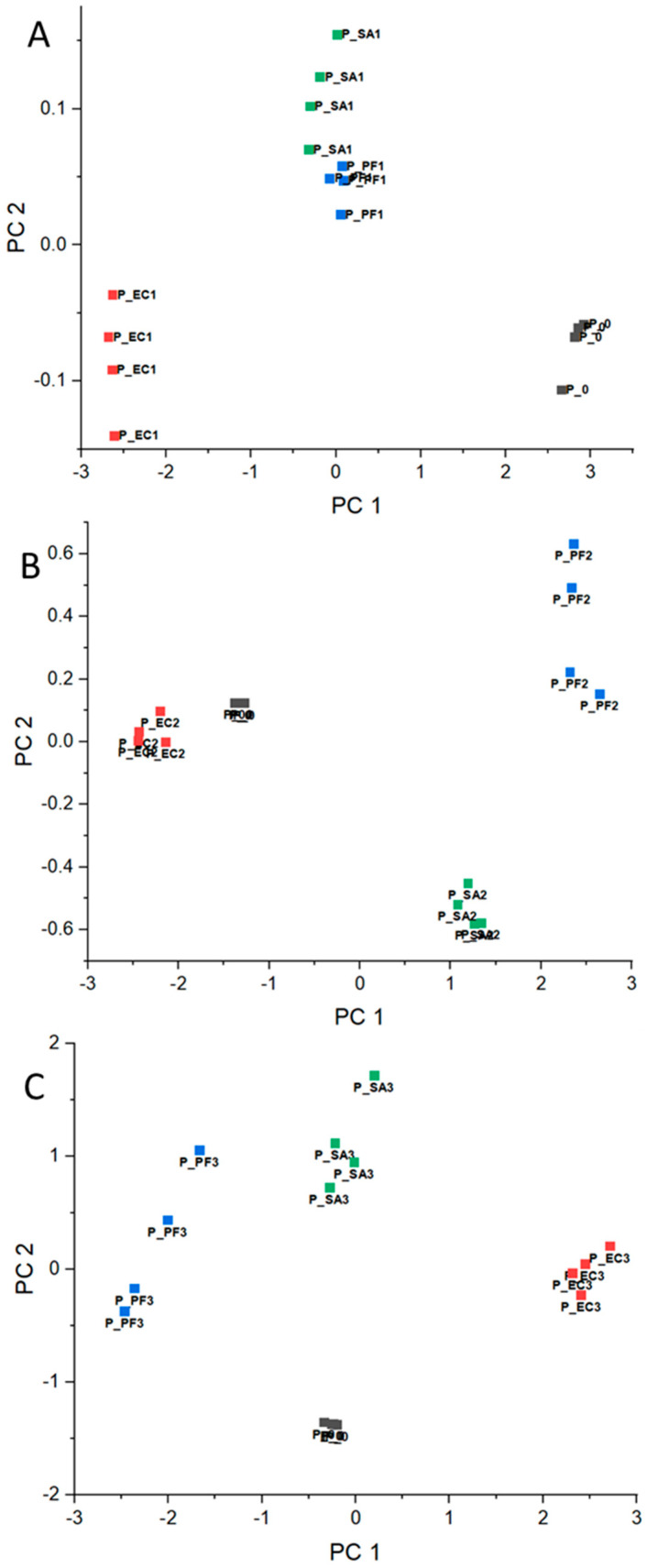
PCA for pork meat without contamination (day 0—■) and inoculated with EC—■, PF—■, and SA—■ for days (**A**) 1, (**B**) 2, and (**C**) 3.

Based on normalized RR data, the PCA corresponding to each experiment day was calculated and presented in Figure 2A–C for beef meat, Figure 3A–C for chicken, and Figure 4A–C for pork. Concerning the chemical composition, there are significant differences in the beef, chicken, and pork meats, for example, protein, moisture, and lipid content [38]. In this way, as mentioned in Section 2.4, the comparison of the results was conducted considering the meats separately and data corresponding to day 0 (before the bacteria inoculation) for each meat as an internal reference throughout the experiment.

Although PCA and other graphical approaches are useful for discrimination analyses, they are based on subjective criteria and are limited to a small amount of data and defined clusters, being arduous and inaccurate their application for data settings containing intersections and overlapping regions. To overcome this issue, automated machine learning classifiers were evaluated for the discrimination of bacteria in the different meats, using the same normalized RR data set as the input. Considering the data from beef, chicken, and pork inoculated with EC, PF, and SA on days 1, 2, and 3, the accuracy obtained for classifiers LDA, IBK, and LMT is presented in Table 1.

In addition to the accuracy, it is also essential to know how the classifier attributed the data among the different classes and for what classes there were mistakes, such as misclassified data. All of this information can be obtained from the confusion matrix. Table 2 summarizes the confusion matrices obtained for all experimental combinations evaluated in this study (meat x day x classifier). For example, considering beef on day 1, we can observe that the same accuracy, 93.8%, was obtained for all classifiers; however, the sample distribution varied, since the classifiers are based on different strategies for discriminating the data. In this way, for LDA and LMT, 1 data point from EC was erroneously classified as SA, while, using IBk, 1 data point from SA was taken as EC. Additionally, since an accuracy of 100% indicates no mistake in the classification, the correspondent confusion matrix is the same regardless of the sample and the classifier.

### 3.3. Bacteria Growing Pattern

**Figure 5:**
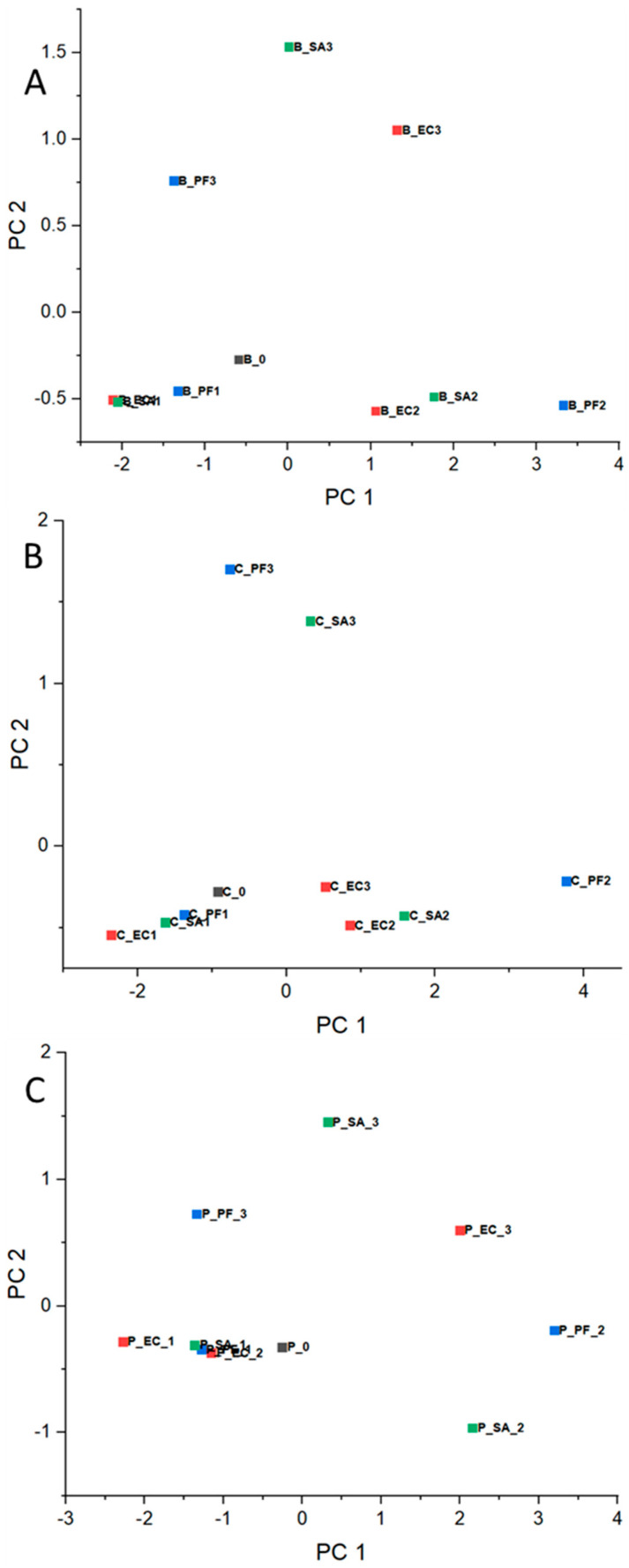
PCA representation based on means of normalized RR data for meat without contamination (day 0—■) and inoculated with EC—■, PF—■, and SA—■ for (**A**) beef, (**B**) chicken, and (**C**) pork.

In order to compare how the bacteria behave over the experiment time in the different meats, the mean values of normalized RR data were taken (Supplementary File Table S2) and used for PCA calculation, as shown in Figure 5A, B, and C for beef, chicken, and pork, respectively.

**Figure 6:**
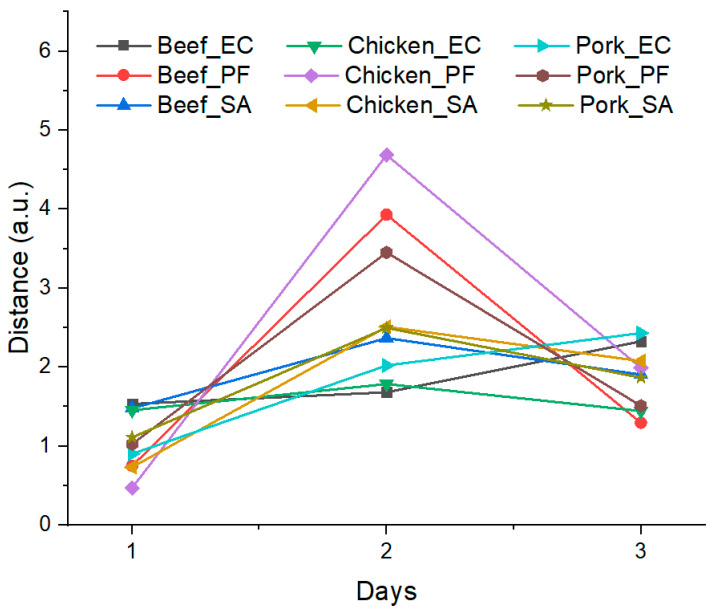
Distance values obtained from samples over experiment time to blank samples on day 0, considering PCAs’ coordinates from Figure 5A–C.

From a graphical analysis overview, it is possible to observe that regardless of the bacteria, the points were shifting away from the blank between days 1 and 2 for all considered meats. This trend can be evaluated by calculating the distance in the Cartesian plane between a given point and the blank using their PCA coordinates, Equation (1). The distances between each point and its corresponding blank are presented in Figure 6.

## 4. Discussion

As shown in Figure 1, the exposition of the sensors to VOCs from the samples led to an increase in the sensors’ conductance. Additionally, differences between the signals for the beef before (day 0) and after (day 1) bacteria inoculation can be observed. These changes can be related to the variation in the composition, the quantity of VOCs, or both, thus it depends on the microorganism’s metabolism, and these can be used as criteria for bacteria differentiation based on the RR of each sample.

In general, PCAs corresponding to day 1 (Figure 2A, Figure 3A, and Figure 4A) presented clusters close to each other for all considered meats, even in an intersection between EC and SA for beef. These results are expected, since the difficulty in differentiating bacteria through VOCs released by them for the shortest period of the experiment is probably related to the intrinsic and extrinsic factors that condition microbial growth, such as the metabolic pathways of microorganisms, the composition of each protein matrix, and the incubation temperature (which is not optimal for the three groups). Furthermore, day 1 may still be a period of adaptation (lag), as will be discussed below with reference to bacteria growing pattern.

In the opposite way, for day 2, the clusters became more distant among them for all considered samples (Figure 2B, Figure 3B, and Figure 4B) and remained for day 3 (Figure 2C, Figure 3C, and Figure 4C). These observations are consistent with the hypothesis that VOCs exhaled by the different bacteria are specific in some manner, and the e-nose was able to differentiate the samples based on the VOC patterns.

In the context of this study, the accuracy is the hit rate of the classifier, which is the amount of successful classified data overall, i.e., an accuracy of 93.8% means that the classifier correctly attributed 15 of the 16 specimens and missed 1; Table 1. In the same way, an accuracy of 100% indicates that all considered samples were correctly classified as they were attributed.

From Table 1 and Table 2, it is possible to state that, in general, the accuracy for all classifiers was considerably high throughout the experiment, achieving 100% accuracy for at least two of the classifiers for each meat after day 2, suggesting that the proposed methodology based on e-nose use has potential for the analysis of microbial contamination in meat.

Concerning the bacteria growing pattern, from Figure 6, it is possible to note that regardless of the bacteria and meat, the distances on day 1 were similar. These data are consistent with the clusters overlap/intersections observed in the PCA and the mistaken attribution in the confusion matrices in the first stages of the experiment. Furthermore, it is possible to correlate the distances obtained for day 1 with the general models for bacteria metabolism and intrinsic and extrinsic factors that affect their growth, as, in the lag phase, bacterial populations are adjusting to a new environment before their exponential growth [12].

For day 2, there was a distinguished, moderate, and slight increase in the distance for PF, SA, and EC, respectively, which suggests a different growth rate between the microorganisms and is consistent with literature data, with *Pseudomonas* growing faster than Enterobacteriaceae in pork meat at 22 °C under an environmental atmosphere [39], while it was observed that *Salmonella* spp. developed at higher rates than *E. coli* on ground beef at 22 °C and in open-air conditions [40].

Finally, for day 3, the calculated distances for EC and SA were similar to day 2, indicating small changes in the VOC patterns. However, a significant decrease in the distance values was obtained for PF regardless of the meat considered, which could be related to the saturation of growth media, nutrient depletion, inhibitory product releasing, or even cellular death by toxic metabolites [12].

## 5. Conclusions

This work demonstrates the application of an e-nose for the direct differentiation of *Salmonella* spp., *E. coli*, and *P. fluorescens* in raw beef, chicken, and pork meats using PCA as a strategy for graphical analysis discrimination, while LDA, IBk, and LMT were used as automated classifier algorithms.

In general, the PCA results showed some proximity between the clusters for day 1, while for days 2 and 3, the clusters were more separated, indicating that the e-nose can discriminate the samples based on VOCs exhaled by the bacteria, and that these VOCs are related to the bacteria growth in the last days of the experiment. Furthermore, from the PCA data, it was possible to estimate the growth of the microorganisms, with an observable pattern between them being independent of the substrate, with variable growth rates in the following increasing order: EC, SA, and PF.

Concerning the automated classifiers, except for the cases of beef and chicken on day 2 with LDA, on which an accuracy of 93,8% was obtained, an accuracy of 100% was obtained for all other conditions on days 2 and 3, suggesting that the proposed methodology based on e-nose has potential for the analysis of microbial contamination in meat.

The proposed methodology based on e-nose use is a simple and low-cost alternative to the traditional microbiological analysis of bacteria in meat, and has the potential for innovation and commercial application since it can be used as a complementary tool in meat quality control, reducing the time, costs, and manipulating steps of the analysis. Further studies have to be performed to optimize and validate this methodology, considering more samples, different incubation periods and temperatures, potential interfering microorganisms, and bacteria quantification to estimate the method’s sensitivity and limit of detection.

## Figures and Tables

**Table 1:** Accuracy values obtained by the different classifiers throughout the experiment.

Meat	Day	Classifier/Accuracy (%)		
		LDA	IBk	LMT
Beef	1	93.8	93.8	93.8
	2	93.8	100	100
	3	100	100	100
Chicken	1	100	100	100
	2	93.8	100	100
	3	100	100	100
Pork	1	100	93.8	75
	2	100	100	100
	3	100	100	100

**Table 2:** Confusion matrices obtained using the different classifiers throughout the experiment.

Classifier(s)/Day	Meat		B_0	B-EC_1	B-PF_1	B-SA_1
LDA/1	Beef	B_0	4	0	0	0
LMT/1		B-EC_1	0	3	0	1
Accuracy (%)		B-PF_1	0	0	4	0
93.8		B-SA_1	0	0	0	4
**Classifier(s)/Day**	**Meat**		**B_0**	**B-EC_1**	**B-PF_1**	**B-SA_1**
IBk/1	Beef	B_0	4	0	0	0
Accuracy (%)		B-EC_1	0	4	0	0
93.8		B-PF_1	0	0	4	0
		B-SA_1	0	1	0	3
**Classifier(s)/Day**	**Meat**		**B_0**	**B-EC_2**	**B-PF_2**	**B-SA_2**
LDA/2	Beef	B_0	4	0	0	0
Accuracy (%)		B-EC_2	0	4	0	0
93.8		B-PF_2	0	0	4	0
		B-SA_2	0	1	0	3
**Classifier(s)/Day**	**Meat**		**C_0**	**C-EC_1**	**C-PF_1**	**C-SA_1**
LDA/2	Chicken	C_0	4	0	0	0
Accuracy (%)		C-EC_1	0	4	0	0
93.8		C-PF_1	0	0	3	1
		C-SA_1	0	0	0	4
**Classifier(s)/Day**	**Meat**		**P_0**	**P-EC_1**	**P-PF_1**	**P-SA_1**
IBk/1	Pork	P_0	4	0	0	0
Accuracy (%)		P-EC_1	0	4	0	0
93.8		P-PF_1	0	0	4	0
		P-SA_1	0	0	1	3
**Accuracy (%)**	**Meat**		**Blank**	**EC**	**PF**	**SA**
100	Bovine	Blank	4	0	0	0
	Chicken	EC	0	4	0	0
	Pork	PF	0	0	4	0
		SA	0	0	0	4

## Data Availability

The data presented in this study are available on request from the corresponding author.

## Supplementary Materials

The following supporting information can be downloaded at: https://www.mdpi.com/article/10.3390/microorganisms12112250/s1, Table S1: Normalized RR data; Table S2: Mean of normalized RR data.
