# Supplementary material for: Direct Discrimination and Growth Estimation of Foodborne Bacteria in Raw Meat Using Electronic Nose
Source: Microorganisms. 2024 Nov 7;12(11):2250. doi: 10.3390/microorganisms12112250 (PMC11596726; doi:10.3390/microorganisms12112250)
Supplement: Supplementary file 1 [file microorganisms-12-02250-s001.zip › microorganisms-3233214-supplementary.pdf]

The RR data presented below are normalized, i. e. it was obtained as the difference of the RR data for a given sample/day to the mean of the RR data of the same sample on the previous day.

For example, B_EC 1 normalized = B_EC 1 - mean of B_0*; B_EC 2 normalized = B_EC 2 - mean of B_EC 1; B_EC 3 normalized = B_EC 3 - mean of B_EC 2

* As B_0 corresponds to the first obtained data and the sample before inoculation, these values were taken integrally and used as an internal reference.

Table 1: Normalized RR data

Beef				
Sample	RR-Sensor 1	RR-Sensor 2	RR-Sensor 3	RR-Sensor 4
B_0	0.0873	0.0767	0.1027	0.1167
B_0	0.0870	0.0777	0.1033	0.1163
B_0	0.0860	0.0757	0.1010	0.1120
B_0	0.0857	0.0743	0.0997	0.1143
B_EC1	-0.0585	-0.0351	-0.0677	-0.0788
B_EC1	-0.0585	-0.0381	-0.0677	-0.0808
B_EC1	-0.0555	-0.0391	-0.0667	-0.0788
B_EC1	-0.0565	-0.0391	-0.0637	-0.0738
B_EC2	0.2105	0.1309	0.3633	0.3502
B_EC2	0.2005	0.1309	0.3503	0.3582
B_EC2	0.2225	0.1439	0.3833	0.3732
B_EC2	0.2235	0.1479	0.4103	0.4042
B_EC3	0.2605	0.2739	0.2953	0.1012
B_EC3	0.3565	0.3659	0.3543	0.1192
B_EC3	0.3425	0.2819	0.3623	0.1192
B_EC3	0.3185	0.3389	0.3023	0.1172
B_PF1	0.0185	0.0099	0.0333	0.0262
B_PF1	0.0205	0.0129	0.0333	0.0262
B_PF1	0.0175	0.0119	0.0293	0.0242
B_PF1	0.0165	0.0099	0.0283	0.0142
B_PF2	0.3875	0.2759	0.7353	0.6852
B_PF2	0.3935	0.2669	0.7353	0.6592
B_PF2	0.3915	0.2649	0.6983	0.6352
B_PF2	0.4025	0.2929	0.7393	0.6912
B_PF3	-0.0435	0.1559	-0.0267	-0.0768
B_PF3	-0.0445	0.1659	-0.0387	-0.0758
B_PF3	-0.0465	0.1809	-0.0317	-0.0818
B_PF3	-0.0475	0.1949	-0.0257	-0.0738
B_SA1	-0.0475	-0.0321	-0.0547	-0.0688
B_SA1	-0.0505	-0.0361	-0.0597	-0.0708
B_SA1	-0.0515	-0.0371	-0.0547	-0.0728
B_SA1	-0.0515	-0.0411	-0.0617	-0.0728
B_SA2	0.2775	0.1969	0.4783	0.4612
B_SA2	0.3015	0.2009	0.5123	0.4742
B_SA2	0.2465	0.1729	0.4353	0.4302
B_SA2	0.2705	0.1819	0.4933	0.4412
B_SA3	0.1235	0.3209	0.0823	-0.0288
B_SA3	0.1215	0.3159	0.0843	-0.0168
B_SA3	0.1215	0.4079	0.0903	0.0172
B_SA3	0.1215	0.2869	0.0973	0.0092

Chicken				
Sample	RR-Sensor 1	RR-Sensor 2	RR-Sensor 3	RR-Sensor 4
C_0	0.0787	0.0697	0.0950	0.1037
C_0	0.0793	0.0683	0.0947	0.1050
C_0	0.0773	0.0650	0.0910	0.1007
C_0	0.0773	0.0673	0.0920	0.1023
C_EC1	-0.0552	-0.0456	-0.0562	-0.0749
C_EC1	-0.0532	-0.0396	-0.0632	-0.0689
C_EC1	-0.0582	-0.0436	-0.0682	-0.0769
C_EC1	-0.0582	-0.0396	-0.0682	-0.0789
C_EC2	0.2208	0.1514	0.3768	0.3971
C_EC2	0.2058	0.1444	0.3768	0.3771
C_EC2	0.2098	0.1404	0.3688	0.3901
C_EC2	0.1978	0.1314	0.3498	0.3531
C_EC3	0.1868	0.1544	0.4138	0.3211
C_EC3	0.1778	0.1624	0.3528	0.2871
C_EC3	0.1608	0.1614	0.3068	0.3231
C_EC3	0.1408	0.1454	0.2838	0.2541
C_PF1	0.0368	0.0254	0.0538	0.0491
C_PF1	0.0398	0.0254	0.0568	0.0491
C_PF1	0.0378	0.0284	0.0578	0.0511
C_PF1	0.0268	0.0224	0.0468	0.0381
C_PF2	0.4248	0.3194	0.8018	0.7821
C_PF2	0.4918	0.3564	0.6758	0.6381
C_PF2	0.4218	0.3084	0.7928	0.7721
C_PF2	0.4828	0.3564	0.7178	0.8551
C_PF3	0.0538	0.2844	0.0158	-0.0279
C_PF3	0.0468	0.4974	0.0288	-0.0579
C_PF3	0.0388	0.3694	0.0198	-0.0369
C_PF3	0.0288	0.2154	0.0238	-0.0389
C_SA1	0.0168	0.0074	0.0228	0.0141
C_SA1	0.0118	0.0034	0.0128	0.0091
C_SA1	0.0138	0.0054	0.0208	0.0131
C_SA1	0.0148	0.0034	0.0348	0.0141
C_SA2	0.3408	0.1644	0.4018	0.4041
C_SA2	0.3638	0.1804	0.4328	0.4351
C_SA2	0.3608	0.1754	0.4388	0.4371
C_SA2	0.3288	0.1584	0.3958	0.4051
C_SA3	0.2278	0.3084	0.1808	0.0301
C_SA3	0.2298	0.3124	0.1688	0.0231
C_SA3	0.2338	0.3444	0.1828	0.0301
C_SA3	0.2378	0.3604	0.1528	0.0491
Pork				
Sample	RR-Sensor 1	RR-Sensor 2	RR-Sensor 3	RR-Sensor 4
P_0	0.0993	0.0833	0.1150	0.1290
P_0	0.0873	0.0777	0.1063	0.1180
P_0	0.0963	0.0817	0.1140	0.1253
P_0	0.0950	0.0807	0.1113	0.1243
P_EC1	-0.0845	-0.0608	-0.0997	-0.1102
P_EC1	-0.0845	-0.0648	-0.0987	-0.1092
P_EC1	-0.0845	-0.0668	-0.1007	-0.1112
P_EC1	-0.0815	-0.0668	-0.0987	-0.1092

P_EC2	0.0085	0.0242	0.0503	0.0278
P_EC2	-0.0045	0.0012	0.0173	0.0138
P_EC2	-0.0035	-0.0028	0.0153	0.0168
P_EC2	0.0225	0.0162	0.0443	0.0498
P_EC3	0.3295	0.3142	0.4683	0.1388
P_EC3	0.3065	0.3222	0.4083	0.1658
P_EC3	0.3335	0.3562	0.4843	0.1598
P_EC3	0.3615	0.2682	0.4533	0.1358
P_PF1	0.0055	0.0012	0.0113	0.0068
P_PF1	0.0085	0.0022	0.0123	0.0058
P_PF1	0.0025	-0.0028	0.0073	-0.0002
P_PF1	0.0075	-0.0008	0.0113	0.0098
P_PF2	0.3785	0.3232	0.7133	0.4508
P_PF2	0.3495	0.3322	0.5553	0.4758
P_PF2	0.3575	0.3552	0.5603	0.4338
P_PF2	0.3695	0.3682	0.5433	0.4208
P_PF3	-0.0325	0.2852	-0.0147	-0.0822
P_PF3	-0.0525	0.1892	-0.0177	-0.0852
P_PF3	-0.0525	0.0892	-0.0417	-0.0932
P_PF3	-0.0605	0.0592	-0.0427	-0.0922
P_SA1	0.0005	-0.0068	-0.0097	-0.0152
P_SA1	0.0015	-0.0078	-0.0067	-0.0152
P_SA1	0.0065	-0.0048	-0.0017	-0.0122
P_SA1	0.0145	-0.0008	0.0063	-0.0032
P_SA2	0.2615	0.1902	0.4943	0.4718
P_SA2	0.2635	0.1852	0.4653	0.4718
P_SA2	0.2635	0.1882	0.4653	0.4378
P_SA2	0.2525	0.1722	0.4713	0.4268
P_SA3	0.1405	0.3402	0.0893	-0.0232
P_SA3	0.1325	0.2922	0.0943	-0.0082
P_SA3	0.1365	0.3402	0.1123	0.0078
P_SA3	0.1385	0.4472	0.1153	-0.0052

Table 2: Mean of normalized RR data

	Beef- RR mean bacteria growth estimation			
	RR-Sensor 1	RR-Sensor 2	RR-Sensor 3	RR-Sensor 4
B_0	0.0865	0.0761	0.1017	0.1148
B_EC1	-0.0573	-0.0378	-0.0664	-0.0781
B_EC2	0.2143	0.1384	0.3768	0.3714
B_EC3	0.3195	0.3152	0.3286	0.1142
B_PF1	0.0183	0.0112	0.0311	0.0227
B_PF2	0.3938	0.2752	0.7271	0.6677
B_PF3	-0.0455	0.1744	-0.0307	-0.0771
B_SA1	-0.0503	-0.0366	-0.0577	-0.0713
B_SA2	0.2740	0.1882	0.4798	0.4517
B_SA3	0.1220	0.3329	0.0886	-0.0048
	Chicken- RR mean for bacteria growth estimation			
	RR-Sensor 1	RR-Sensor 2	RR-Sensor 3	RR-Sensor 4
C_0	0.0782	0.0676	0.0932	0.1029
C_EC1	-0.0562	-0.0421	-0.0639	-0.0749
C_EC2	0.2086	0.1419	0.3681	0.3793
C_EC3	0.1666	0.1559	0.3393	0.2963

C_PF1	0.0353	0.0254	0.0538	0.0468
C_PF2	0.4553	0.3352	0.7471	0.7618
C_PF3	0.0421	0.3417	0.0221	-0.0404
C_SA1	0.0143	0.0049	0.0228	0.0126
C_SA2	0.3486	0.1697	0.4173	0.4203
C_SA3	0.2323	0.3314	0.1713	0.0331
	Pork- RR mean for bacteria growth estimation			
	RR-Sensor 1	RR-Sensor 2	RR-Sensor 3	RR-Sensor 4
P_0	0.0945	0.0808	0.1117	0.1242
P_EC1	-0.0838	-0.0648	-0.0994	-0.1099
C_EC2	0.0058	0.0097	0.0318	0.0271
C_EC3	0.3328	0.3152	0.4536	0.1501
C_PF1	0.0060	-0.0001	0.0106	0.0056
C_PF2	0.3638	0.3447	0.5931	0.4453
C_PF3	-0.0495	0.1557	-0.0292	-0.0882
C_SA1	0.0058	-0.0051	-0.0029	-0.0114
C_SA2	0.2603	0.1839	0.4741	0.4521
C_SA3	0.1370	0.3549	0.1028	-0.0072
